# An Investigation of the Effect of Catecholamines and Glucocorticoids on the Growth and Pathogenicity of *Campylobacter jejuni*

**DOI:** 10.3390/pathogens9070555

**Published:** 2020-07-10

**Authors:** Brendha Truccollo, Paul Whyte, Declan J. Bolton

**Affiliations:** 1 Food Safety Department, Teagasc Food Research Centre, Ashtown, Dublin 15, D15 KN3K Dublin, Ireland; brendha.truccollo@teagasc.ie; 2 School of Veterinary Medicine, University College Dublin, Belfield, Dublin 4, D04 V1W8 Dublin, Ireland; paul.whyte@ucd.ie

**Keywords:** *Campylobacter*, broiler, host stress, catecholamines, glucocorticoids, virulence, pathogenicity

## Abstract

*Campylobacter* spp. are major causes of foodborne illness globally, and are mostly transmitted through the consumption and handling of poultry. *Campylobacter* infections have widely variable outcomes, ranging from mild enteritis to severe illness, which are attributed to host interactions and the virulence of the infecting strain. In this study, in order to investigate the effect of host stress on the growth and pathogenicity of *C. jejuni*, three strains associated with human infection and two strains from broilers were subject to growth, motility, adhesion and invasion assays, in response to exposure to catecholamines; epinephrine, norepinephrine and the glucocorticoid neuroendocrine hormones corticosterone, cortisol and cortisone which are associated with stress in humans and broilers. Catecholamines resulted in significantly increased growth, adhesion and invasion of Caco-2 cells. Corticosterone promoted growth in one of five strains, and cortisone resulted in a significant increase in motility in two out of five strains, while no significant differences were observed with the addition of cortisol. It was concluded that stress-associated hormones, especially catecholamines, may promote growth and virulence in *Campylobacter*.

## 1. Introduction

*Campylobacter* spp. are leading causes of bacterial foodborne illness globally [1]. It is estimated that every year, one in 10 people contract campylobacteriosis globally [2]. In the European Union (EU), over 200,000 cases are reported annually, however it is estimated that the real incidence is closer to 9 million cases per annum [3]. Additionally, campylobacteriosis costs the EU EUR 2.4 billion annually in healthcare costs and lost working days [3]. Most infections are caused by *Campylobacter jejuni*, accounting for over 90% of cases [4]. Other species, including *C. coli*, *C. lari*, *C. upsaliensis* and *C. fetus*, are also implicated in human illness, however, these are less frequently recovered [5].

*C. jejuni* is usually acquired by humans through the handling and consumption of raw or undercooked contaminated poultry [6]. *Campylobacter* is generally transmitted to broilers horizontally, after 14 days of age, and often rapidly spreads to an entire flock [7]. *C. jejuni* colonizes the cecum of broilers, which consists of a pair of blind pouches above the ileum where fine particulates are deposited for prolonged digestion [8]. *Campylobacter* spp. persist in broilers until slaughter, and processing practices, including evisceration, contribute to the contamination of broiler meat, posing a risk of exposure to consumers [9]. Human campylobacteriosis occurs through colonization of the colon following ingestion of an infectious dose of *Campylobacter* cells [1]. Patients may experience symptoms within 48 h following exposure, which include malaise; abdominal cramps that can resemble appendicitis; fever; nausea, which may be followed by vomiting; and watery or hemorrhagic diarrhea [1,10,11]. Symptoms generally subside without requiring medical intervention after 3 to 7 days [12], however, recrudescence several weeks after the infection had been cleared was previously reported in a healthy host [13]. Serious complications can occur in a subset of cases, including acute peripheral neuropathies such as Guillain-Barré Syndrome and Miller Fisher Syndrome, translocation to critical organs in the event of bacteremia, septicemia or meningitis, gastrointestinal disorders including inflammatory bowel disease (IBD) or irritable bowel syndrome (IBS), and autoimmune illnesses, such as reactive arthritis [1]. Previous reports have also described an association between *Campylobacter* spp. infection and colon cancer [14].

Understanding the diverse manifestation of *Campylobacter* illness and its ability to persist in the harsh poultry environment and be rapidly transmitted to thousands of birds remains elusive, as it is a notoriously fastidious organism with limited virulence and survival factors compared to other pathogens. Associations between host stress and infection leading to more severe illness have been previously reported [15,16,17]. Indeed, it has been suggested that stress and a prior gastrointestinal infection are considered significant risk factors for the development of IBS, and stress may contribute to the development of septicemia, following bacterial infection [18,19]. Host stress results in the activation of the hypothalamic-pituitary-adrenal axis, leading to the increased production of stress neuroendocrine hormones, including catecholamines and glucocorticoids, which can enter the gastrointestinal tract, coming into contact with the local microbiome [20]. Previous studies have shown that catecholamines including epinephrine and norepinephrine have resulted in increased pathogenicity in a number of bacteria, including *Salmonella* spp., *Escherichia coli*, *Yersinia* spp., *Vibrio* spp. and *Campylobacter* spp. [21]. The interaction between the gut microbiome and host-derived neuroendocrine hormones is further substantiated by the bacterial production of hormones, including norepinephrine, GABA, serotonin, dopamine and somatostatin by gut microbiota including *Bacillus* spp., *Lactobacillus* spp., *Serratia* spp., *Escherichia* spp. and *Enterococcus* spp. [22]. In contrast, limited knowledge is available on the effect of stress-associated glucocorticoids, such as corticosterone, cortisol and cortisone, on their ability to interact with bacteria, though it has been previously reported that probiotic administration of *Lactobacillus helveticus* and *Bifidobacterium longum* promoted reduced levels of corticosterone in mice, and the bacterial modification of cortisol has been previously reported [22,23], which supports the possibility that gut pathogens interact with glucocorticoid stress hormones.

The pathogenicity of *Campylobacter* spp. depends on functions such as chemotaxis, motility, adhesion and invasion of host epithelial cells, cytotoxin production, among other important factors, including capsule and biofilm formation, lipooligosaccharide class variation, secretion systems and vesicle formation, and various colonization factors that contribute to *Campylobacter* spp. survival, transmission and infection symptoms in symptomatic hosts [4,24,25]. During host colonization, motility enables *Campylobacter* spp. to travel to the colonization site and through the intestinal mucosa, where it is able to proliferate following attachment to host cells, followed by internalization into intestinal epithelial cells and the secretion of the cytolethal distending toxin (CDT), which leads to host cell apoptosis. Hence, mutations in genes involved in motility, adhesion and invasion have been previously associated with decreased host colonization [26,27]. Furthermore, the combination of these putative virulence factors and the host’s inflammatory response results in the observed symptoms in *Campylobacter* enteritis [1].

The objective of this study was to investigate the effect of catecholamines; epinephrine and norepinephrine, and glucocorticoids; corticosterone, cortisol and cortisone on *C. jejuni* growth, motility, and its ability to adhere to and invade Caco-2 cells, to expand the current knowledge on the association between host stress and *C. jejuni* infection.

## 2. Results

### 2.1. Bacterial Growth

Five isolates were selected for this study, three of human clinical origin and two from broilers (Table 1). *C. jejuni* NCTC 11168 was selected for this study as it is a well characterized strain that has been previously used in similar studies [15,16,17], while wild-type strains associated with human illness and broilers were included to ensure the strains tested were more representative of those currently found in nature. 

**Figure 1:**
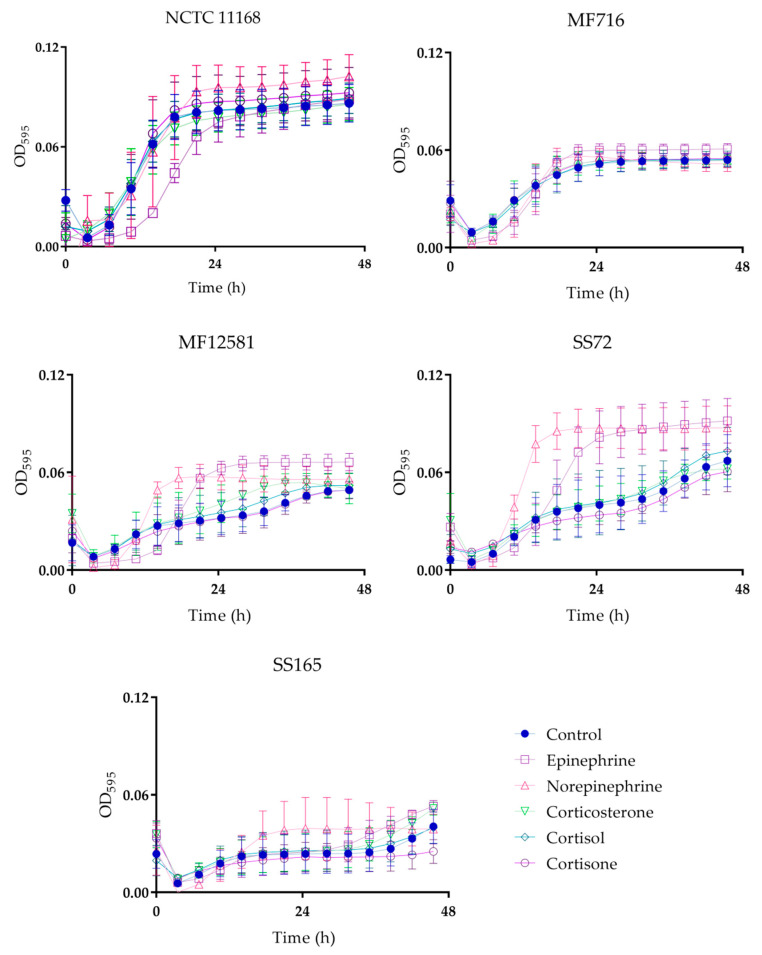
Growth (OD_595_) of *C. jejuni* in response to 100 µM of catecholamines and glucocorticoids. Line plots represent the mean of three replicates with standard error.

In this study, the presence of both catecholamines and glucocorticoids affected *Campylobacter* growth (Figure 1). The addition of norepinephrine promoted the growth of four out of five isolates, which was significant in NCTC 11168 at the stationary phase of growth (*p* < 0.01), and in MF12581 and SS72 at the log and stationary phases of growth (*p* < 0.05 and *p* < 0.001, respectively). A log phase delay with the addition of norepinephrine was observed in MF716 (*p* < 0.05), followed by an increase in OD_595_ at the end of the log phase of growth as compared to the control, but this was not statistically significant (*p* > 0.05). Increased growth was observed in four out of five isolates in response to epinephrine, which was significant in MF716 at the stationary phase of growth *(p* < 0.05), and in MF12581 and SS72 at the log and stationary phases of growth (*p* < 0.001). Log phase delays were observed with the addition of epinephrine in NCTC 11168 (*p* < 0.01), MF716 (*p* < 0.05), and MF12581 (*p* < 0.05), which were subsequently followed by increased growth for MF716 and MF12581.

The addition of glucocorticoids did not significantly affect growth except for an increase in MF12581 with the addition of corticosterone at the stationary phase of growth (*p* < 0.05), although small increases were observed in NCTC 11168, MF12581 and SS72 with the addition of cortisol or cortisone, and small decreases in growth with the addition of cortisone were observed in SS72 and SS165, but none of these were significant (*p* > 0.05).

### 2.2. Motility

**Figure 2:**
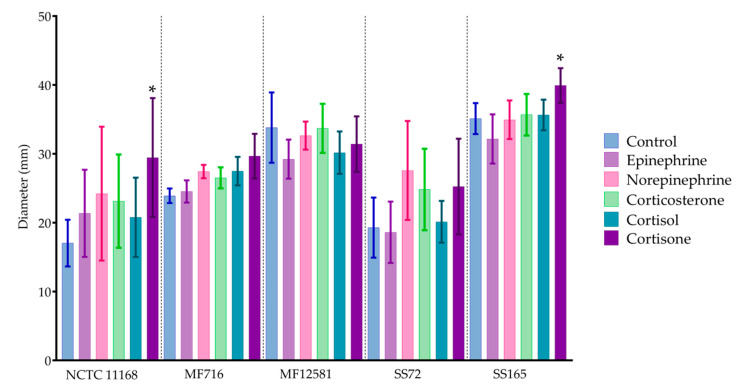
Motility of *C. jejuni* in response to catecholamines and glucocorticoids. Bars represent the mean of four replicates with standard error, where * = *p* < 0.05.

The overall motility of each isolate was significantly different from one another (*p* < 0.05), as shown in Figure 2. However, no significant differences in motility were detected in response to each hormone treatment, with the exception of cortisone, which significantly promoted motility in NCTC 11168 and SS165 by 12.4 mm and by 4.8 mm, respectively (*p* < 0.05). No significant effect was observed with the addition of catecholamines, corticosterone or cortisol (*p* > 0.05). 

### 2.3. Adhesion and Invasion of Caco-2 Cells

**Figure 3:**
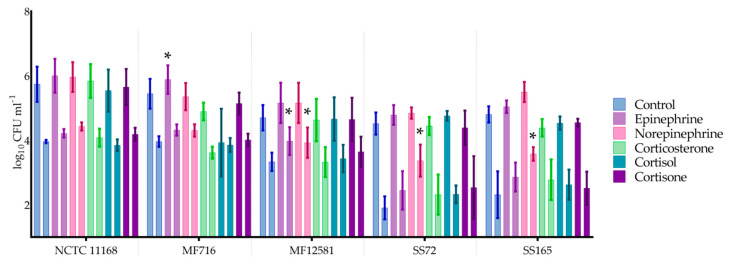
*C. jejuni* adhesion and invasion of Caco-2 cells in response to pre-treatment with catecholamines and glucocorticoids. The first bar within each test represents mean adhesion counts, followed by mean invasion counts. Each bar represents the mean of four replicates with standard error, where * = *p* < 0.05.

Overall, catecholamines promoted both adhesion and invasion across the isolates tested, while the response to glucocorticoids showed some variation. Furthermore, the human isolates had higher adhesion and invasion than the broiler isolates (*p* < 0.05; Figure 3).

Marginally increased adhesion was observed across all isolates in the presence of catecholamines (*p* > 0.05), with the exception of norepinephrine treated MF716. The presence of epinephrine resulted in a significant increase in adhesion in MF716 by 0.44 log_10_ colony forming units (CFU) mL^−1^ (*p* < 0.05).

Increased invasion was detected in all isolates, in response to catecholamines. Increased internalization by 0.6, 1.46 and 1.25 log_10_ CFU mL^-1^ was observed in response to norepinephrine in MF12581, SS72 and SS165, respectively, which were statistically significant (*p* < 0.05).

Overall, decreased adhesion to Caco-2 cells was observed in the presence of all three glucocorticoids tested, which was not statistically significant (*p* > 0.05). In contrast, increased internalization was observed in the presence of glucocorticoids on average by 0.13 (0.14 SEM), 0.12 (0.11 SEM) and 0.28 (0.10 SEM) log_10_ CFU mL^−1^ with corticosterone, cortisol and cortisone, respectively. Notably, however, the presence of glucocorticoids did not result in statistically significant differences in either adhesion or invasion (*p* > 0.05).

### 2.4. Expression of Virulence Genes

**Figure 4:**
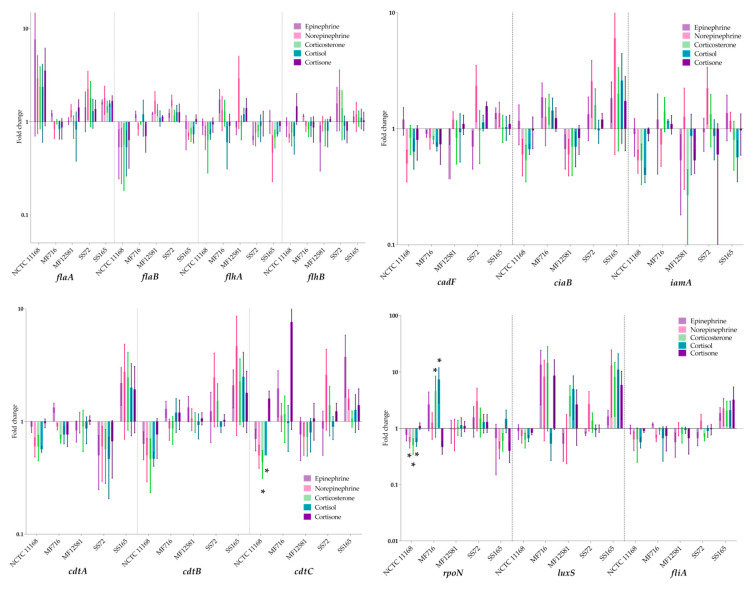
Fold-change of the expression of genes associated with motility (*flaA*, *flaB*, *flhA*, *flhB*), adhesion and invasion (*cadF*, *ciaB*, *iamA*), cytolethal distending toxin (CDT) synthesis (*cdtA*, *cdtB*, *cdtC*), and transcriptional regulators (*rpoN*, *fliA*, and *luxS*), in response to cultivation with catecholamines and glucocorticoids normalized to the expression of *16S rRNA*. Each bar represents the mean of three replicates with standard error, where * = *p* < 0.05.

The expression of genes associated with motility (*flaA*, *flaB*, *flhA* and *flhB*), CDT production (*cdtA*, *cdtB* and *cdtB*), adhesion and invasion (*cadF*, *ciaB* and *iamA*) and transcription regulators (*fliA*, *rpoN* and *luxS*) was examined in *C. jejuni*, in response to cultivation with catecholamines and glucocorticoid neuroendocrine hormones. The resulting fold changes are shown in Figure 4. The expression of two genes was significantly altered: *cdtC* was significantly down-regulated in the presence of corticosterone and cortisol in NCTC 11168 by 0.4- and 0.5-fold, respectively (*p* < 0.05). Additionally, *rpoN* was significantly down-regulated in response to norepinephrine, corticosterone and cortisol in NCTC 11168 by 0.6-, 0.5- and 0.6-fold, respectively (*p* < 0.05). Lastly, it was up-regulated in the presence of cortisol in MF716 by 7.6-fold, and down-regulated in the presence of cortisone by 0.5-fold. No significant change in the expression of the remaining genes tested was observed (*p* > 0.05). The mean expression observed in genes associated with motility, adhesion or invasion reflected some of the significant phenotypic findings. However, the lack of significance in the expression observed suggests that other genes not included in this panel may be more involved in the observed phenotypic response.

## 3. Discussion

In this study, we investigated the effect of catecholamines and glucocorticoids on the growth, motility, adhesion and invasion of *C. jejuni*. The presence of catecholamines resulted in a significant increase in growth for most isolates, and in the increased adhesion of Caco-2 cells for MF716, and in the increased invasion of Caco-2 cells for MF12581, SS72 and SS165, respectively. Similar results have been previously reported [15,16,17]. Increased growth was observed in response to epinephrine and norepinephrine, which has been widely reported [15,16,17]. In contrast to Xu et al. (2015), who observed increased growth in NCTC 11168 with the addition of epinephrine, the opposite effect was observed in our study. However, epinephrine did promote growth in three other strains in this study, which supports the previous report that epinephrine promotes growth in *C. jejuni* strains [17]. 

Significantly increased motility in the presence of norepinephrine has also been previously reported [15,16], but was not observed in this study. This is possibly due to variations in assay conditions, as motility had been previously measured in basal media [16] and in iron-restricted nutrient-rich media [15], while, in this study, a semi-solid nutrient-rich agar was used. In the latter report, no difference in *C. jejuni* motility was observed, with the addition of norepinephrine in nutrient-rich media that was not iron-restricted, which is in agreement with the observations in this study [15]. Additionally, in this study adhesion to and invasion of epithelial cells were promoted following pre-incubation with catecholamines, which is in agreement with previous studies [15,16,17]. Previous reports show that *C*. *jejuni* can survive and replicate within epithelial cells [28], and internalization can protect *C. jejuni* from immune responses, impairing disease clearance [29].

Taken together, these results indicate that epinephrine and norepinephrine promote the growth of *C. jejuni*, and enhanced pathogenicity in the form of increased adhesion and invasion of epithelial tissue may also be observed with certain isolates. However, no significant difference in gene expression was observed in response to either treatment in this study, with the exception of the downregulation of *rpoN* in one isolate in the presence of norepinephrine (NCTC 11168). Similar results were reported previously for the expression of the motility, adhesion and invasion-associated genes examined in response to epinephrine and norepinephrine [17]. This indicates that the observed effects on *C. jejuni* growth and adhesion in response to catecholamines are possibly due to other genes and proteins that were not included in this panel. A previously proposed mechanism for growth promotion in *C. jejuni* and other pathogens consists of catecholamine-mediated iron-uptake, as the catechol moiety of epinephrine and norepinephrine exhibits high affinity for ferric iron [15,17,30]. This was supported by a previous study, where the FeEnt receptor CfrA was involved in norepinephrine utilization by *C. jejuni* [17,31]. In contrast, it was reported that iron uptake genes *cfrA*, *chuA* and *Cj0178* were not up-regulated in the presence of epinephrine nor norepinephrine [17]. Notably, the possibility that catecholamines promote *C. jejuni* pathogenicity through other means cannot be disregarded. Indeed, the two-component regulator gene *Cj1608*, the sensor kinase gene *Cj1492c* and the outer membrane porin *Omp50* were significantly up-regulated in response to catecholamine exposure in the same study, which could have implications on the modulation of *C. jejuni*’s pathogenicity in response to epinephrine and norepinephrine [17]. 

To the best of our knowledge, there are no previous reports on the effect of glucocorticoids on the growth and pathogenicity of *C. jejuni*. In order to gain a broader perspective on the effect of host stress on the growth and pathogenicity of pathogens of public health relevance, it is important to characterize their response to a wide range of stress-derived neuroendocrine hormones that pathogens may be exposed to at their colonization site. In humans, cortisol is mostly secreted during stress, while, in reptiles and birds, corticosterone is more abundantly found in circulation [32]. In this study, *C. jejuni* exposure to glucocorticoids resulted in small increases in pathogenicity. No significant increase in adhesion or invasion to Caco-2 cells was detected in response to any of the three treatments, however, a significant increase in growth was observed in response to corticosterone in one isolate, and cortisone resulted in a significant increase in motility in two of the five isolates. Overall, it appears that glucocorticoids can promote growth and virulence in *C. jejuni*, but not to the same degree as catecholamines. In epithelial cells, glucocorticoids are internalized, leading to the transcription of target genes and the activation of glucocorticoid signaling pathways, resulting in physiological effects, including blood glucose regulation, reduced bone remodelling, immunosuppression, and the secretion of catecholamines epinephrine and norepinephrine [33,34]. The mechanism behind glucocorticoid-mediated growth and motility in *C. jejuni* requires further investigation, as no significant changes in the expression of the genes included in this study were observed in isolates that displayed a significant phenotypic response.

In previous studies, cortisol was associated with increased growth of *Porphyromonas gingivalis* [35] and altered colony morphology of *Flavobacterium columnare* in vitro [36]. In vivo, they are associated with increased virulence of the oral microbiome [37], and invasive and more severe infection with other pathogens in humans [38,39,40], which may be partly attributed to glucocorticoid-mediated immunosuppression. Furthermore, an organism that can often be found in the gastrointestinal tract of animals, *Clostridium scindens*, was previously found to synthesize enzymes that can cleave the side-chain of glucocorticoids, such as cortisol, resulting in the formation of androgens in the gastrointestinal tract, which can then be reintroduced into circulation [23]. These reports indicate that there may be an underexplored relationship between the direct effects of glucocorticoids on the pathogenicity of microorganisms of public health relevance. In this study, glucocorticoids resulted in small increases in motility and growth, however, this was not observed in all isolates tested, suggesting that *C. jejuni* responds to glucocorticoids in a strain-specific manner that may lead to increased proliferation and/or motility.

No significant differences in the expression of genes associated with motility (*flaA*, *flaB*, *flhA*, *flhB*), cytotoxin (*cdtA*, *cdtB*), adhesion and invasion (*cadF*, *ciaB*, *iamA*), or virulence gene regulators (*luxS*, *fliA*) were observed in response to each treatment. Notably, the expression of *rpoN* was significantly down-regulated with norepinephrine, corticosterone and cortisol in NCTC 11168, and it was significantly down-regulated in the presence of cortisone and up-regulated in the presence of cortisol in MF716. *rpoN* regulates the transcription of flagellar genes in *C. jejuni* [4]. However, in this study, changes in the expression of *rpoN* did not coincide with significant changes in the expression or other motility genes or to phenotypic motility.

Furthermore, *cdtC* was significantly down-regulated in NCTC 11168 in the presence of corticosterone and cortisol. The effect of host stress neuroendocrine hormones on CDT production has not been widely studied in *Campylobacter* spp. However, previous reports based on the strain NCTC 11168 indicate that no increases in CDT synthesis occurs in the presence of catecholamines [15,17]. In contrast, the non-significant up-regulation of *cdtA*, *B* and *C* in response to all treatments in one of the five isolates indicates that there may be a relationship between host stress and CDT production, and it may be strain-specific.

*luxS*, which is involved in signal transduction, also exhibited high levels of up-regulation that were not statistically significant, which could potentially be implicated in the organism’s response to catecholamines and glucocorticoids. LuxS synthesises autoinducer-2 which is involved in quorum sensing and physiological pathways including biofilm formation, autoagglutination, CDT expression, flagellar expression, oxidative stress and colonization [41]. Furthermore, it has been previously found to be involved in the response to epinephrine in enterohaemorrhagic *E. coli* and *Actinobacillus pleuropneumoniae* [42,43].

Overall, catecholamines promoted growth, adhesion and invasion in most *C. jejuni* strains in this study, while some increases in growth and motility were observed with glucocorticoids, indicating that the host stress response may result in increased *C. jejuni* proliferation and pathogenicity. In addition to increased pathogenicity, these neuroendocrine hormones also remodulate host metabolic and immunological activity, resulting in immunosuppression, which can contribute to longer or more severe disease outcomes [33,44]. *C. jejuni* that were pre-treated with norepinephrine previously exhibited increased translocation to the liver in chicks [16]. Additionally, stress and corticosterone administration in broilers was previously found to significantly alter their microbiota composition, including the proliferation and virulence of other pathogens including *Salmonella* spp. [45,46]. Other studies reported that stressful conditions for broilers, including partial depopulation and transportation, result in the increased shedding of fecal campyobacters, which can promote their spread to adjacent birds, contributing to the previously reported rapid spread throughout an entire flock, following the initial introduction [47,48]. The significance of this interaction for broilers is that stress may promote the spread of *Campylobacter* spp. during rearing in response to unfavorable conditions and low broiler welfare. On the other hand, for the human population host stress could potentially promote a more severe disease outcome following *C. jejuni* infection. Indeed, stress has been described as a major risk factor to the onset of more severe illness following microbial infection [18,19]. Further investigation into the mechanisms underlying these interactions and into possible in vivo ramifications would be beneficial to further elucidate the link between host stress and microbial illness.

## 4. Materials and Methods 

### 4.1. Bacterial Growth Conditions

Purified isolates (Table 1) that were previously confirmed by PCR [5] were recovered from defibrinated horse blood at −80 °C and cultivated in modified charcoal cefoperazone agar (mCCDA, Oxoid, Basingstoke, UK), supplemented with mCCDA selective supplement (SR0155E, Oxoid, Basingstoke, UK) at 37 °C for 48 h microaerobically. They were subsequently sub-cultured onto the chosen inoculation medium for each assay and incubated for 48 h under the same conditions.

### 4.2. Bacterial Growth Assay

The bacterial growth assay was performed based on previous reports [15,16]. Each isolate was cultivated microaerobically at 37 °C on Columbia Blood Agar (CBA, Oxoid, Basingstoke, UK) supplemented with 5% defibrinated horse blood for 48 h, prior to inoculation. Dulbecco’s Modified Eagles Medium (DMEM, Invitrogen, Renfrew, UK) supplemented with 1% MEM Non-Essential Amino Acids (MEM NEAA), 10% foetal bovine serum, 4 mM glutamine and with or without 100 µM of epinephrine, norepinephrine, corticosterone, cortisol or cortisone, whereby the concentration was based on previous studies with norepinephrine [15], was inoculated with 10^4^ cfu mL^−1^ of *C. jejuni* and dispensed into a U-bottom 96-well plate at a volume of 200 µL. The 96-well plate was placed in a microaerobic atmosphere (10% CO_2_, 5% H_2_, 5% O_2_, balancing N_2_) for 30 min, and was then sealed with a clear and air-tight film to preserve the microaerophilic atmosphere, followed by incubation in a plate reader (Multiskan FC, ThermoFisher, UK), at 37 °C for 48 h. OD_595_ readings were taken every 30 min. 

### 4.3. Motility Assay

Cultures from CBA were resuspended in maximum recovery diluent (MRD, Oxoid, Basingstoke, UK) to reach 0.1 OD_595_, and then 10 µL from each suspension was dispensed onto soft brain heart infusion agar (0.4% agar), supplemented with or without 100 µM of each hormone that was dissolved in 1 mL of 1% Tween 80 solution, before being incorporated with the agar, while the control also received 1 mL of 1% Tween 80. Once dry, the samples were incubated at 37 °C for 48 h in a microaerobic atmosphere (CampyGen, Oxoid, Basingstoke, UK). The diameter of each growth zone was measured after incubation.

### 4.4. Adhesion and Invasion Assay

#### 4.4.1. Cultivation of Caco-2 Cells

Caco-2 cultivation and maintenance were carried out as previously described [16,49]. Caco-2 cells stored in DMSO_4_ at a concentration of 1 × 10^6^ cells were centrifuged and resuspended in 15 mL of DMEM with 1% MEM NEAA, 10% foetal bovine serum, 4 mM glutamine and incubated at 37 °C for 5 days to reach 80% confluency. The medium was changed every 2–3 days. The cells were then dispensed into T75 flasks and incubated at 37 °C for 72 h, before seeding into a 24-well plate at 3 × 10^5^ cells/well. The wells were incubated for 11 days to reach a total cell concentration of 7 × 10^7^ cells/well. DMEM supplemented with 100 IU/ml of penicillin-streptomycin was used to feed the cells every 3–4 days until 3 days before infection, when it was replaced with fresh DMEM. On day 11, the cells were washed with Dulbecco’s phosphate-buffered saline (dPBS; ThermoFisher, Horsham, UK) and 1ml of fresh DMEM was added to the monolayer.

#### 4.4.2. Infection of Caco-2 Cells

The gentamicin protection assay was performed to calculate the number of internalized and adherent cells, as previously described [16,49]. Purified colonies of each isolate were taken from CBA and added to 20 mL of DMEM with or without 100 µM of each hormone. They were then incubated at 37 °C for 48 h. Each inoculum was then centrifuged at 4000× *g* for 10 min, resuspended in 10 mL of phosphate-buffered saline (PBS), re-centrifuged at 4000× *g* for 10 min and resuspended in fresh DMEM. A volume of 0.5 mL from each culture was adjusted to 0.1 OD_600_ and dispensed into each Caco-2 monolayer in the 24-well plate for a multiplicity of infection (MOI) of 3. The 24-well plates were subsequently incubated at 37 °C for 3 h in a microaerobic atmosphere.

#### 4.4.3. Adhesion Assay 

Each monolayer was washed with dPBS to remove unattached *Campylobacter* cells, and then incubated with 1% Triton X-100 for 5 min to lyse the Caco-2 cells, assisted by gentle pipetting. The contents of each well were serially diluted in MRD and then spread onto mCCDA, before all samples were incubated at 42 °C for 48 h in a microaerobic atmosphere.

#### 4.4.4. Invasion Assay

The wells were washed with dPBS and then incubated with DMEM supplemented with gentamicin (100 µg/mL) for 1h at room temperature. After incubation, DMEM-gentamicin was removed, the Caco-2 cells were thoroughly washed with PBS, followed by incubation with 1% Triton X-100 for 5 min. The Caco-2 cells were lysed by gentle pipetting and the remaining mixture was serially diluted in MRD and spread over mCCDA before microaerobic incubation for 48 h at 42 °C.

As the adhesion assay records the total number of cells attached and internalized, to calculate the total number of cells attached the value for internalized cells was subtracted from the adhesion assay values.

### 4.5. Virulence Gene Expression RT-qPCR

#### 4.5.1. Bacterial Growth Conditions

The cultivation of *C. jejuni* for transcriptomic analysis was carried out as previously described [50]. Mueller Hinton Agar (MHA; Oxoid, Basingstoke, UK) was inoculated with each isolate to form a bacterial lawn, which was incubated at 37 °C for 48 h microaerobically. The resulting lawn was resuspended in 1ml of Mueller-Hinton Broth (MHB, Oxoid, Basingstoke, UK), and the optical density was adjusted to 0.3 OD_595_. A total of 85 µL from this suspension was transferred to a biphasic culture flask containing 8 mL of MHA and 8 mL of MHB, which was supplemented with or without 100 µM of each hormone. All samples were incubated at 37 °C microaerobically, until they reached 0.1 OD_595_ (16–20 h) [50].

#### 4.5.2. RNA Extraction, Purification and cDNA Synthesis

RNA extraction and purification was carried out as previously described [49]. The liquid portion from each flask was centrifuged at 4000× *g* for 5 min, decanted, resuspended in 10 mL of phosphate-buffered saline (PBS, Oxoid, Basingstoke, UK), then centrifuged at 4000× *g* for 5 min and decanted before performing the RNA extraction (RNeasy Mini Kit, Qiagen, Manchester, UK). Cell lysis was performed with the addition of 400 µL of 1 mg/mL lysozyme in TE buffer (pH 8.0), and pellets were vortexed every 2 min for 10 min. Buffer RLT (1200 µL) and 14.3 M β-mercaptoethanol (12 µL) were subsequently added to precipitate and preserve RNA in the sample, which was subsequently purified following the manufacturer’s instructions (RNeasy Mini Kit, Qiagen, Manchester, UK). The resulting RNA sample was then purified from any remaining DNA contamination (Turbo DNase Kit, ThermoFisher, UK), following the manufacturer’s instructions. Protein contamination from the previous step was removed by performing a nucleic acid precipitation step by adding 0.1 volumes of sodium acetate (pH 5.2) and 2.5 volumes of ice-cold 100% ethanol to each sample and incubating at −20 °C overnight. Each sample was then centrifuged at 3800× *g* for 15 min, and resuspended in 70% ethanol, vortexed, and centrifuged at 3800× *g* for 8 min. The supernatant was carefully discarded, and the remaining RNA pellet was allowed to fully dry before it was resuspended in 50 µL of nuclease-free H_2_O. Each RNA sample was then standardized and converted to cDNA using the SuperScript Vilo cDNA synthesis kit (ThermoFisher, UK), following the manufacturer’s instructions.

#### 4.5.3. RT-qPCR

The relative quantification of virulence genes was carried out, as previously described [49,51]. A standard curve was generated for each target gene (*16S rRNA*, *flaA*, *flaB*, *flhA*, *flhB*, *cdtA*, *cdtB*, *cdtC*, *cadF*, *ciaB*, *iamA*, *rpoN*, *fliA* and *luxS*) by diluting the starting cDNA sample (0.1 ng/µL) between 0 to 5-fold. Each reaction consisted of 5 µL Sybr Green (Roche Diagnostics, Burgess Hill, UK), 0.5 µL of 10 µM forward and reverse primers, 3 µL nuclease-free water and 0.01 ng/µL of cDNA. Cycling conditions consisted of 1 cycle at 95 °C for 10 min, 45 amplification cycles at 95 °C for 10 s, 55 °C for 30 s and 72 °C for 10 s. A melting curve was generated by incubating the samples between 65 °C and 97 °C, with a ramp rate of 0.11 °C s^−1^ with continuous fluorescence monitoring. All genes were normalized to the expression of the housekeeping gene 16S rRNA based on the methods from Koolman et al., 2016, which was found to be stably expressed.

### 4.6. Statistical Analysis

Bacterial growth assays were analyzed via a two-way ANOVA with Dunnett’s multiple comparisons test on GraphPad Prism version 7.0 (GraphPad Software, San Diego, CA, USA). Motility, adhesion and invasion assays were analyzed via a two-way ANOVA with Tukey’s multiple comparisons test, and gene expression data was analyzed with Friedman’s test on GenStat version 18.1 (VSN International Ltd., Hemel Hempstead, UK). Results where *p* < 0.05 were considered statistically significant.

## 5. Conclusions

In this study, catecholamines effected significantly increased growth and pathogenicity (adhesion and invasion of Caco-2 cells) for most of the isolates tested. These observations, coupled with increased motility with cortisone, suggest that host stress may promote the growth and pathogenicity of certain strains of *C. jejuni*, which has implications in human illness and broiler production that requires further investigation.

## Figures and Tables

**Table 1:** *C. jejuni* isolates used in this study.

Isolate	Source
*C. jejuni* subsp. *jejuni* NCTC 11168	Human
*C. jejuni* MF716	Human
*C. jejuni* MF12581	Human
*C. jejuni* SS72	Broiler
*C. jejuni* SS165	Broiler
